# Driven by Deformable Convolution and Multi-Plane Scale Constraint: A Hazy Image Dehazing–Stitching System

**DOI:** 10.3390/s26051551

**Published:** 2026-03-01

**Authors:** Sheng Hu, Han Xiao, Cong Liu, Haina Song, Min Liu, Liang Li, Hongzhang Liu

**Affiliations:** 1 School of Electrical and Electronic Engineering, Hubei University of Technology, Wuhan 430068, China; 20181008@hbut.edu.cn (S.H.); 102310266@hbut.edu.cn (H.X.); 20141109@hbut.edu.cn (C.L.); songhn_cqupt@163.com (H.S.); liu_min@mail.hbut.edu.cn (M.L.);; 2 School of Nuclear Technology and Chemistry & Biology, Hubei University of Science and Technology, Xianning 437100, China

**Keywords:** image dehazing, image stitching, deep learning, deformable convolution, feature matching

## Abstract

Adverse weather conditions, such as fog, degrade image quality and affect the performance of deep learning-based image processing algorithms, whereas advanced driver assistance systems (ADASs) urgently demand image clarity and large-field-of-view perception in foggy environments. Existing image dehazing methods rarely consider the non-uniform and dense distribution of particles in fog, leading to severe attenuation of background information. Image stitching, owing to the low-brightness and low-texture characteristics of ADAS scenarios and differences between sensors, faces challenges such as difficult feature point extraction and matching and poor stitching quality. To address these issues, this study proposes a non-uniform dehazing method based on Deformable Convolution v4 (DCNv4), designing a DCNv4-based transform-like network to achieve long-range dependence and adaptive spatial aggregation, combined with a lightweight Retinex-inspired Transformer for color correction and structure refinement. Meanwhile, a multi-plane scale constraint module is introduced based on the LightGlue feature matching network to improve matching accuracy and homography matrix estimation precision, and an adaptive fusion stitching method is adopted to eliminate artifacts and transition zones. Experimental results show that the proposed method effectively improves feature matching accuracy and homography matrix calculation precision, achieving Peak Signal-to-Noise Ratios (PSNRs) of 22.78 dB and 24.34 dB on the NH-HAZE and BRAS datasets, respectively, which are superior to those of existing methods. This provides a reliable environmental perception solution for autonomous driving in foggy environments, verifying its effectiveness and practicality.

## 1. Introduction

Images captured under hazy conditions, whether naturally occurring or artificially synthesized, exhibit similar characteristics, such as low visibility, reduced contrast, and degraded structural details. These deteriorated properties severely degrade the performance of various visual tasks, such as object recognition, tracking, and segmentation systems, thereby hindering their application in hazy scenarios. This predicament urgently calls for effective dehazing methods in diverse contexts. In recent years, panoramic driving assistance systems have emerged as cutting-edge driving aid technologies. By stitching and fusing images captured by multiple cameras, such systems provide drivers with a 360-degree blind-spot-free view, effectively compensating for the limitations of traditional driving visibility. However, most existing panoramic driving systems have relatively limited applications under adverse weather conditions. Therefore, developing a panoramic-assisted driving system tailored to foggy environments is crucial.

The core goal of assisted driving is “safety”, and the prerequisite for safety is comprehensive, accurate, and real-time environmental perception. By integrating local views from multiple cameras, image stitching technology constructs a blind-spot-free global perception. Meanwhile, it addresses key challenges, including blind spots, multi-sensor fusion accuracy, adaptability to complex environments, and system computing power optimization, thereby serving as a crucial bridge connecting sensor acquisition and decision-making control.

From the perspective of user experience, 360° panoramic parking and ultra-wide-field-of-view cruising supported by image stitching directly enhance driving safety and convenience of driving. From the perspective of technological evolution, the views generated by image stitching provide core technical support for the transition from advanced assisted driving (AAD) to autonomous driving (AD).

Recent advances in image dehazing have been significantly influenced by the application of deep learning technologies, a development driven by their tremendous success in fields such as classification and object detection. Notably, compared with the previously dominant atmospheric scattering model (ASM)-based framework [1,2,3,4], deep learning-based methods have demonstrated superior performance in removing haze from images with complex and spatially varying haze distributions, emerging as the primary approach to address non-uniform dehazing challenges. Recent research has mainly focused on exploring robust and powerful representation learning mechanisms to model the mapping between foggy and fog-free images. These methods typically learn spatial and frequency representations simultaneously, integrate specialized architectures to expand the receptive field, or leverage large-scale convolutional neural networks (CNNs) [5,6,7] pre-trained on massive datasets to capitalize on the benefits of transfer learning. For instance, DWT-FFC [8] employs a discrete wavelet transform to effectively capture spatial and spectral information, adopts fast Fourier convolution (FFC) to enhance receptive capability, and utilizes a pre-trained ConvNeXt model to facilitate transfer learning; DehazeFormer [9] introduces a Transformer-based architecture with a shifted window partitioning scheme based on reflection padding for dehazing tasks.

Nevertheless, current methods face several challenges that require further exploration. First, traditional convolutional neural networks (CNNs) often suffer from strict inductive biases, and CNN-based models frequently rely on relatively large fixed dense kernels (e.g., 31 × 31) to facilitate robust representation learning. This strategy incurs significant computational overhead and lacks the ability to adapt to input conditions for adaptive spatial aggregation. Second, although Transformer-based architectures can capture long-range dependencies and promote adaptive spatial aggregation, they are hindered by inefficient computation and memory usage. The inherent complexity of the self-attention mechanism scales quadratically with the input resolution, which precludes its application in high-resolution dehazing scenarios.

To address these challenges, this study proposes the EDCT module, a novel non-uniform dehazing method based on a deformable convolution Transformer architecture. The model consists of two primary components: a dehazing and a refinement module. A Transformer-like dehazing branch is designed within the dehazing module, which incorporates multiple EDCN blocks. Unlike traditional Transformer architectures, the EDCN block leverages Deformable Convolution v4 (DCNv4) instead of the standard self-attention mechanism, thereby enabling the model to benefit from long-range dependency modeling and adaptive spatial aggregation capabilities. Furthermore, by eliminating redundant operations in conventional DCN (i.e., softmax normalization in spatial aggregation), the EDCN architecture achieves faster convergence and inference speed. In the refinement module, we formulated a lightweight Retinex-inspired Transformer for color correction and structural refinement. Through these specialized designs, the EDCT module can achieve consistency in color and detail.

The core bottleneck of traditional image stitching methods lies in their limited feature representation capability. For instance, handcrafted features, such as SIFT, essentially capture local grayscale variations or edge structures of images. When encountering complex environments (e.g., fog-induced image blurriness, insufficient illumination at night, or roads with repetitive textures), a large number of feature points are either lost or mismatched, resulting in misalignment during stitching. Existing methods automatically learn features via deep learning (e.g., convolutional neural networks (CNNs) and Transformers) without the need for manually designed rules. Traditional image stitching methods, which are centered on “handcrafted features + geometric models,” are suitable for static and simple environments. Their advantages include low computational complexity and cost; however, they suffer from limited robustness and scene adaptability. In contrast, existing methods, rooted in “deep learning + data-driven” paradigms, overcome the bottlenecks of traditional approaches through the autonomous learning of high-level features, dynamic adaptation to view variations, and semantic-level fusion. They can meet the full-scene requirements of assisted driving, ranging from low- to high-speed scenarios and simple to complex environments.

To better perceive the surrounding situation during driving and reduce the occurrence of hazardous accidents, we propose and implement a feature matching method based on a multi-plane scale constraint for dehazed images. To address issues such as obvious transition zones and artifacts in image stitching, we developed an adaptive fusion stitching method. Finally, we validated the effectiveness of the proposed method for acquiring panoramic traffic images under foggy conditions.

Our main contributions are as follows:

1. We propose an effective non-uniform dehazing method based on a deformable convolution Transformer, followed by a Retinex-inspired Transformer network for color and detail refinement.
2. We designed a DCNv4-based Transformer-like network for dehazing, which provides long-range dependency modeling and adaptive spatial aggregation capabilities while demonstrating faster convergence and inference speed.
3. We developed a multi-plane scale constraint-based image matching method and an adaptive fusion stitching algorithm. Combined with the dehazing module, this study provides a comprehensive solution for surrounding perception in assisted driving under foggy conditions.

## 2. Related Work

### 2.1. Transformer-Based Image Dehazing

Owing to their flexibility in capturing global dependencies, Transformers have achieved remarkable success in various computer vision tasks. For instance, Uformer [10], SwinIR [11], and Restormer [12] have demonstrated state-of-the-art (SOTA) performance in numerous image restoration tasks. In recent years, a variety of methods have been proposed to apply Transformers to single-image dehazing. Based on Swin Transformer, Song et al. [13] developed DehazeFormer, which incorporates improved normalization layers, activation functions, and spatial information aggregation mechanisms. Guo et al. [14] proposed a hybrid framework that combines Transformers with CNNs for image dehazing via transmission-aware 3D positional embedding and feature modulation. Qiu et al. [15] applied Taylor expansion to approximate traditional softmax attention in Transformers, achieving linear complexity while retaining the flexibility of global dependency modeling. These methods have exhibited SOTA performance on various dehazing benchmark datasets, inspiring us to incorporate an improved Transformer architecture into our proposed model.

### 2.2. Deep Learning-Based Image Stitching

Building upon traditional feature point matching algorithms, researchers have gradually incorporated adaptive mechanisms and deep learning technologies to further enhance image stitching performance. In 2017, Yu and Koltun [16] proposed Dilated Residual Networks (DRNs), an innovation that significantly advanced feature extraction in image stitching technology. DRNs primarily introduce dilated convolutions to expand the receptive field of convolutional neural networks (CNNs) while maintaining controllable computational complexity. This approach addresses the limitations of traditional CNNs when processing wide-field-of-view images, enabling the extraction of richer contextual information during image stitching. Subsequently, in the same year, Wang et al. [17] proposed an improved feature point detection and matching method based on classic algorithms such as SIFT and SURF. In 2018, Nguyen et al. [18] developed an unsupervised learning-based deep homography estimation model, aiming for fast and robust image stitching. In 2020, Nie et al. [19] presented a view-free image stitching network based on global homography, specifically designed for image stitching tasks in complex scenarios. Unlike traditional methods that typically rely on local feature point matching or view transformation, Nie et al.’s approach innovatively addresses stitching challenges through global homography, eliminating view constraints. In 2021, Zhao et al. [20] proposed a novel image stitching method via deep homography estimation, which significantly improved stitching accuracy and efficiency. By leveraging deep learning models to automatically estimate homography matrices between images, this research greatly simplified the feature matching steps in traditional stitching processes, offering a novel perspective on image matching.

### 2.3. Image Stitching Method Based on Multi-Plane Scale Constraint Matching

To address the problem of large feature matching errors and failure to achieve accurate registration caused by illumination changes, lens distortion, and projection errors in image stitching tasks, DeTone et al. [21] proposed a deep learning-based homography estimation method. By constructing a neural network that takes paired images as input, the method calculates the homography by estimating the changes in four vertices in the images, directly outputting the transformation matrix in an end-to-end manner. Liu et al. [22] proposed a novel detector-free feature matching method called GeoFormer. Based on the matching results obtained from local feature matching methods, GeoFormer performs region-of-interest (ROI) search using the classic Random Sample Consensus (RANSAC) algorithm to obtain the homography matrix. It then computes cross-attention in a centralized manner to enhance local features and achieve correct matches.

## 3. Proposed Method

**Figure 1:**
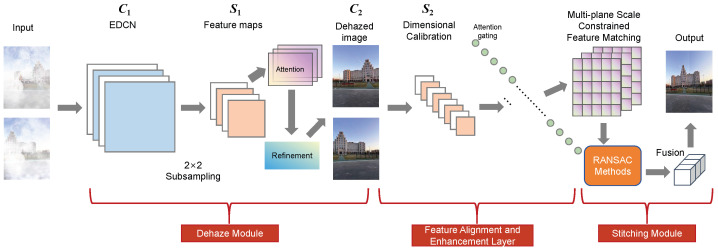
Overall system framework diagram.

Our primary work involves designing a fusion system for image dehazing and stitching, designed to assist surrounding perception in assisted driving. The training process is divided into two phases. In Phase 1, we propose a novel dehazing module based on DCNv4 (Deformable Convolution v4) and adopt a lightweight Transformer for color and detail enhancement (refinement module) to achieve preliminary dehazing. In Phase 2, the refinement module is integrated into the optimization process for detailed refinement. After image dehazing, the dehazed images proceed to the next processing stage. We analyze feature matching networks and develop a multi-plane scale constraint matching method: by constructing several local planes, we identify point pairs with high consistency within each local plane to form a new feature set, enabling accurate feature matching. Based on the precise matches, the RANSAC algorithm is employed to solve for the transformation matrix. To address potential issues such as transition zones and artifacts in image stitching, we propose an adaptive fusion stitching method that introduces an adaptive weighting function to guide the stitching process, effectively eliminating prominent transition zones. The overall system framework is illustrated in Figure 1.

The system adopts a hybrid data flow design combining serial and parallel processing, which not only ensures the coherence of processing logic but also improves computational efficiency. Instead of directly feeding the output of the dehazing module into the stitching module, an intermediate adaptation is performed through a feature alignment and enhancement layer. This layer calibrates the dimensions of the dehazed image features, mapping 3-channel image features into 128-dimensional feature vectors to match the output dimension of the feature extraction in the stitching module. Meanwhile, it screens key features through an attention gate to suppress invalid information such as fog residues and noise, reducing the computational redundancy of the stitching module.

The feature extraction unit of the stitching module shares the bottom convolutional layers (Conv1–Conv3) with the dehazing module. Leveraging the foggy image feature representation capability learned from the dehazing task, the number of model parameters is reduced. Additionally, when the matching error is large, the refinement module enhances the detail extraction of low-texture regions to improve the matchability of feature points.

### 3.1. Dehazing Module Branch

#### 3.1.1. EDCN Module

**Figure 2:**
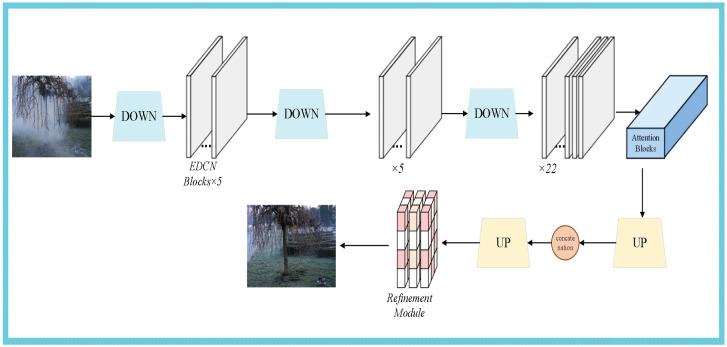
Overall framework of the EDCN dehazing method.

Given an input image $I\in \{R^{W\times H\times 3}\}$ in the dehazing network, it is encoded into $F_{i}\in R^{\frac{W}{4\times 2^{i}}\times \frac{W}{4\times 2^{i}}\times d_{i}}$ via downsampling, where W, H, and $d_{i}$ denote the image width, image height, and dimension of latent features, respectively. After each downsampling step, several EDCN blocks—sharing a similar architecture with conventional Transformer blocks—are adopted for representation learning. However, the core operator of our EDCN is not the global attention mechanism of Transformers, but Deformable Convolution v4 (DCNv4), which is realized by removing the softmax normalization operation in DCNv3. The method framework is illustrated in Figure 2.

Given each feature $F_{i}$ and the current pixel $p_{0}$, the formula for DCNv4 is expressed as

(1) $$y\left(p_{0}\right)=\sum_{g=1}^{G}\sum_{k=1}^{K}w_{g}m_{gk}x_{g}\left(p_{0}+p_{k}+\Delta p_{gk}\right)$$

where *K* denotes the total number of sampling points, and *G* represents the total number of aggregation groups. For the *g*-th group, $w_{g}$ is the positional projection weight of the group, with $d_{i}^{\prime }=\frac{d_{i}}{G}$ denoting the group dimension, and $m_{gk}\in R$ is the modulation scalar for the *k*-th sampling point in the *g*-th group, without normalization along dimension *K* via the softmax function. In this experiment, the number of aggregation groups *G* is set to 4 (verified by experiments: G=4 achieves the optimal balance between performance and computational efficiency). The grouping is based on the channel correlation of the feature map, ensuring that features within each group have similar semantic information to improve aggregation efficiency. $x_{g}$ denotes the sliced input feature map, corresponding to the offset $\Delta p_{gk}$ of the grid sampling position in the *g*-th group. A 3×3 convolution kernel sampling point configuration is adopted with K=9. The initial spatial coordinates of the sampling points are $p_{k}=\left\{\left(i,j\right)|i,j\in \left\{-1,0,1\right\}\right\}$ (relative to the convolution kernel center). During training, the offset $\Delta p_{gk}=\left(\Delta p_{gk,x},\Delta p_{gk,y}\right)$ is predicted by an independent 1×1 convolution layer, and its value range is constrained within [−1,1] to ensure that the sampling points do not deviate too far from the target area.

**Figure 3:**
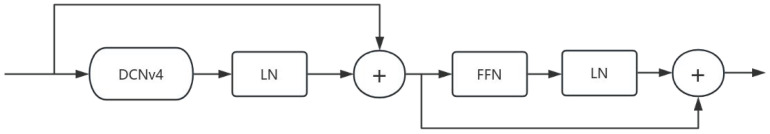
EDCN module framework diagram.

Unlike DCNv3, which normalizes the modulation scalar $m_{gk}$ using the softmax operation, we do not incorporate any normalization function in the EDCN to achieve unbounded dynamic weights for the modulation scalars. This helps achieve faster convergence and inference speed compared to DCNv3, conventional convolutions, and attention blocks in Transformers. Furthermore, the core operator in our EDCN only adopts a 3 × 3 kernel to learn long-range dependencies, which is easier to optimize compared to large kernels. Notably, although the parameters of our dehazing module are relatively large, the EDCN module enables our model to effectively perform high-resolution dehazing without special designs for high-resolution images (e.g., 4000 × 6000). The EDCN module is illustrated in Figure 3.

#### 3.1.2. Dehazing Module Loss Function

The loss function adopted to optimize the dehazing module is expressed as

(2) $$L_{loss}=L_{1}+\alpha L_{SSIM}+\beta L_{Percep}$$

Herein, $L_{1}$ and $L_{SSIM}$ denote the $L_{1}$ loss and SSIM loss. Additionally, α and β are hyperparameters, set to 0.4 and 0.01, respectively.

#### 3.1.3. Transformer-Based Refinement Module

Based on Retinex theory, we first compute the channel-wise mean value for each pixel of the input image; this mean value is then integrated with the global attention mechanism of the Transformer to construct a two-stage framework of coarse estimation and fine correction, which is designed to rapidly capture prior information regarding the global illumination distribution. Specifically, our refinement module initially predicts the illumination map and corresponding illumination features to recover color and detail information lost due to fog scattering; subsequent iterative refinement is further implemented via the Transformer-based refinement module, with the final illumination component formulated as follows:

(3) $$L_{\mathrm{final}}=L_{\mathrm{coarse}}\cdot \sigma \left(\mathrm{Attention}(L_{\mathrm{coarse}},R_{\mathrm{init}})\right)$$

Herein, $R_{\mathrm{init}}=I/L_{\mathrm{coarse}}$ denotes the initial reflection component; Attention(·) represents the sliding window self-attention mechanism (window size: 8×8), which is used to capture the local variations in illumination; and σ(·) denotes the Sigmoid function, which dynamically adjusts the local intensity of the illumination component to avoid halo artifacts. Thus, our final dehazed image can be obtained as follows:

(4) $$I_{dehazed}=\phi \left(\theta \left(I_{hazy}\right),mean\left(\theta \left(I_{hazy}\right)\right)\right)$$

Herein, θ and ϕ denote the dehazing module and the refinement module, respectively. The core of the Retinex algorithm is based on the image illumination–reflectance decomposition model, with its fundamental assumption being that an image can be expressed as the product of the scene reflectance component (reflectance) and the illumination component (illumination). The image I(x,y) perceived by the observer is composed of the incident light image L(x,y) and the reflectance image R(x,y). That is, incident light irradiates a reflective object, and the reflected light generated by the object’s reflection enters the human eye, thereby forming the observed image. The fundamental formula is expressed as

(5) I(x,y)=R(x,y)×L(x,y)

Herein, L(x,y) denotes the observed image, R(x,y) represents the object’s reflectance characteristics (related to color), and L(x,y) denotes the incident scene illumination distribution. The goal of the Retinex algorithm is to eliminate the impact of uneven illumination and recover true reflectance information by separating L(x,y) and R(x,y), thereby achieving color correction and detail enhancement.

The illumination distribution of foggy images exhibits global smoothness: the scattering effect of fog particles renders the illumination variation gentler compared to haze-free images; thus, the channel-wise mean can serve as an effective prior. The self-attention weight is defined as

(6) $$A_{i,j}=\exp\left(-\frac{∥L_{\mathrm{coarse}}\left(i\right)-L_{\mathrm{coarse}}{\left(j\right)∥}^{2}}{2\sigma^{2}}\right)$$

With this weight, targeted correction is applied to illumination mutation regions to suppress halo artifacts.

### 3.2. Image Stitching Module Branch

#### 3.2.1. Feature Extraction Based on Hybrid Attention Mechanism

Image features refer to structures or information that can represent the characteristics of images, mainly including shape, color, texture, etc. Image feature point extraction is a critical issue in image stitching tasks, as the quality of feature points directly affects subsequent matching operations. This paper adopts a hybrid attention mechanism structure to highlight the information of regions of interest (ROIs) in feature maps from both spatial and channel dimensions, thereby improving the accuracy of feature extraction.

In image processing tasks, incorporating attention information enables more effective extraction of key information. The principle of the attention mechanism lies in focusing more on regions of interest: it generates a weight through neural network operations, where the weight indicates the importance of the current region of interest, and selectively highlights salient features based on varying degrees of importance. Commonly used attention networks can be categorized into three types: spatial attention mechanisms (e.g., STNs [23] (Spatial Transform Networks)), channel attention mechanisms (e.g., SENet (Squeeze-and-Excitation Networks)), and hybrid attention mechanisms (e.g., CBAM [24] (Convolutional Block Attention Module)).

**Figure 4:**
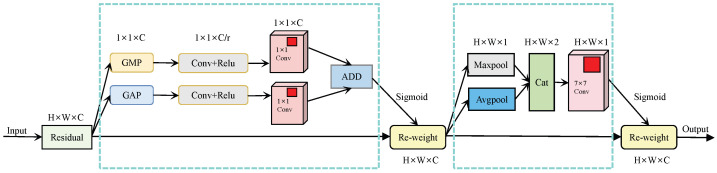
Network architecture diagram of CBAM.

CBAM consists of two components: channel attention and spatial attention. The network architecture is shown in Figure 4. The feature input is denoted as $F\in R^{C\times H\times W}$. CBAM operates through sequential processing to obtain the channel attention feature $M_{c}\in R^{c\times 1\times 1}$ and the spatial attention feature $M_{s}\in R^{1\times H\times W}$. The network execution flow is as follows:

(7) $$F^{\prime }=M_{c}\left(F\right)⨂F$$

(8) $$F^{\prime \prime }=M_{s}\left(F^{\prime }\right)⨂F^{\prime }$$

Herein, ⨂ denotes the element-wise multiplication operation. In this operation, the weights are broadcast accordingly based on their respective dimensions: channel weights are broadcast spatially, while spatial weights are broadcast across channels. After the input features are fed into the entire network, the channel attention operation is performed first. Global average pooling and global max pooling are applied to aggregate information from the feature maps spatially, yielding features $F_{avg}^{c}$ and $F_{max}^{c}$. These two features are then fed into a multilayer perceptron (MLP), which outputs two distinct channel features. Finally, element-wise addition is performed, followed by processing with an activation function to obtain the output feature vector. The process is as follows:

(9) $$M_{c}\left(F\right)=\delta \left(W_{2}\cdot \sigma \left(W_{1}\cdot F_{\mathrm{avg}}^{c}+b_{1}\right)+b_{2}+W_{4}\cdot \sigma \left(W_{3}\cdot F_{\max}^{c}+b_{3}\right)+b_{4}\right)$$

Equation (9) defines the channel attention mechanism for feature refinement, where $M_{c}\left(F\right)$ denotes the channel attention map generated from the input feature map *F* (dimension: C×H×W). The activation function δ(·) represents the Sigmoid function, which normalizes the attention map values to the range [0,1] to weight channel importance, while σ(·) denotes the ReLU activation function introduced to add nonlinearity in the multilayer perceptron (MLP) layers. Specifically, $W_{1}$ and $W_{3}$ are the first-layer weight matrices (dimension: C/r×C, *r* = channel reduction ratio) of the MLP for the average pooling and max pooling branches. $W_{2}$ and $W_{4}$ are the corresponding second-layer weight matrices. The terms $b_{1},b_{3}$ and $b_{2},b_{4}$ represent the first-layer and second-layer bias vectors (dimension: C/r×1 and C×1) for the two branches.

**Figure 5:**
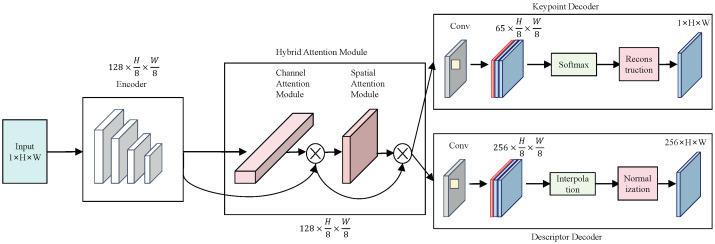
Network structure diagram incorporating hybrid attention mechanism.

The STN attention mechanism and SENet attention mechanism only focus on feature enhancement in either the spatial or channel dimension, failing to consider information from the other dimension. Considering that the information in the feature maps of the original network is equally important in both spatial and channel dimensions, this paper adopts a hybrid attention method to balance feature information from both dimensions, thereby enhancing the network’s feature extraction capability, as shown in Figure 5. In terms of network structure, due to the need to integrate both types of information, the hybrid attention module should be placed at an intermediate position in the network architecture to avoid unreasonable configurations of feature map size or number of channels. The hybrid attention module is positioned after the encoder based on the feature hierarchy and gradient stability principles. Shallow features before the encoder lack global semantics and contain excessive noise, which would bias attention learning, while encoder output features provide stable and semantically complete representations. This arrangement ensures reliable attention modeling and stable network optimization. Through network structure analysis, the hybrid attention module is positioned after the encoder. After processing by the encoder, the output feature maps have 128 channels and a spatial size of 1/8 of the original image, where both the channel and spatial dimensions are moderate, enabling the module to exert a more significant effect.

#### 3.2.2. Multi-Plane Scale Constraint Feature Matching Method

After acquiring high-quality feature points, the image stitching task requires two additional modules: image registration and image fusion. Currently, most traditional methods achieve matching using the Euclidean distance or Hamming distance of descriptors, and can be combined with deep learning-based feature point extraction methods to achieve moderate performance. However, due to their relative simplicity, these methods exhibit poor performance in slightly complex scenarios with low matching accuracy.

Therefore, to address the problem of significant feature matching errors caused by illumination variations, projection errors, and other factors in image stitching—which prevent accurate registration—this chapter develops and implements a multi-plane scale constraint matching method based on the LightGlue [25] feature matching network. By introducing a multi-plane scale constraint module to adjust the distribution of feature points, the feature points become more globally distributed, improving matching precision and reducing homography matrix estimation errors. Additionally, this chapter completes image stitching using an improved fusion stitching algorithm, eliminating artifacts, transition zones, and other potential issues in image stitching to enhance visual quality.

The ability of a feature matching network to accurately complete feature matching tasks is largely determined by the network input: paired input feature points with better global distribution result in superior network matching performance. This chapter adopts a multi-plane scale constraint method, assuming that an image consists of multiple local planes satisfying affine consistency conditions. The image is divided into multiple planes of different scales, and points with high consistency in each plane region are retained to form a new feature set. This approach alters the distribution of feature points, avoids excessive concentration of local feature points, and achieves more accurate feature matching.

1. Spatial Feature Point Analysis

For images of two views captured in 3D space, their homography transformation can be approximately represented by an affine transformation in space. To achieve this transformation, a prior condition must be satisfied: the matched point pairs must maintain high geometric consistency. However, the basic assumptions of planarity and correct projection are often difficult to meet in actual images, and can be violated by various practical factors.

First, the actually captured image is a 2D projection of a 3D space, serving as a tangent plane of the 3D surface. There exists a certain offset between a point on this tangent plane and the real surface, leading to nonlinear deviations in the projection of all 3D points not lying on the tangent plane. The distance between the projected points and the actual surface points exhibits a nonlinear relationship, and this deviation becomes more pronounced with increasing curvature. Second, some of the matched key points may not represent identical projections—a common issue in images captured from different viewpoints in real scenes. Illumination variations can easily affect the localization of key points, thereby preventing the matching from producing the same transformation relationship. Finally, distortions caused by camera lenses can also introduce nonlinear relationships between points in the two view images. These factors increase the difficulty of registration and lead to larger subsequent homography estimation errors. To minimize the errors caused by the aforementioned factors, this chapter develops and implements a multi-plane scale constraint method. By constructing multiple local planes and applying constraints within each local plane, a new feature set is formed to complete accurate feature matching.

2. Local Plane Construction and Constraints

Multi-plane scale constraint mainly consists of two steps: local plane construction and local plane constraint. The local plane construction method involves first assigning a confidence score to each keypoint, where the confidence score is determined by the ratio of distances to the nearest-neighbor keypoints. Next, extreme points with the highest confidence scores in local regions are identified. If a point is an extreme point in its region, this region is designated as a local plane region, and the point is used to guide the consistency search of points within the region. Through this approach, local plane construction is completed while ensuring the uniqueness and coverage of the identified extreme points.

After constructing several local planes, local plane constraints need to be applied. First, the correspondences between different local planes in the image pair are established. Subsequently, different constraint spaces are built based on these local spatial correspondences to complete consistency searches. Affine transformation can be regarded as a good approximation of the local transformation around a point and can be used for local plane constraints. Near-neighbor correspondences are utilized to guide the search for points in local images, aiming to select a limited set of well-distributed points as guides for local transformations and search for consistent correspondences around them.

Specifically, if the seed point pair $x_{1}$ and $x_{2}$ corresponds to a pair of matched extreme points, the corresponding similarity transformation can be derived from the local plane features. This transformation consists of a scale component $\sigma^{s_{i}}$ and an orientation component $\alpha^{s_{i}}$, as expressed in the following formula:

(10) $$\begin{array}{c} \alpha^{s_{i}}=\alpha_{2}^{s_{i}}-\alpha_{1}^{s_{i}} \end{array}$$

(11) $$\begin{array}{c} \sigma^{s_{i}}=\sigma_{2}^{s_{i}}-\sigma_{1}^{s_{i}} \end{array}$$

Herein, $S_{i}$ denotes the seed point pair between Plane 1 and Plane 2. All point pairs in this local plane feature pair are verified, and those satisfying affine transformation consistency are stored in set M. Considering the existence of errors, an orientation component threshold and a scale component threshold are set as the criteria for scale consistency between candidate point pairs and the seed point pair. If the threshold conditions are met, the candidate point pairs are deemed to satisfy the consistency requirement.

Filtering out outliers by setting a fixed threshold for residuals fails to adequately consider the connections between different planes. Instead, by defining a statistical significance threshold for the inlier set, we do not restrict the deviation between the affine model and the true model, thereby improving the quality and accuracy of the data to obtain a more reliable feature point set. The specific step involves mapping the residual values $r_{k}$ in the residual set R to measurement confidence scores $c_{k}$, as expressed in the following formula:

(12) $$c_{k}\left(R\right)=\frac{T}{\left|R\right|\cdot \frac{r_{k}^{2}}{R_{2}^{2}}}$$

**Figure 6:**
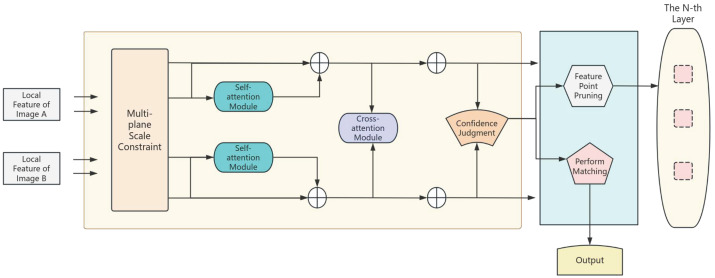
Multi-plane scale constraint-based feature matching network structure diagram.

Herein, *R* denotes the total number of elements in the residual set, and *T* represents the number of inliers (i.e., the count of samples with errors less than $r_{k}$). This confidence score $c_{k}$ indicates the ratio of the number of inliers to the number of correct samples potentially identified under discrete statistics. A high threshold $t_{c}$ is applied to constrain the measurement confidence scores, ensuring that the data is presented with high confidence. Similar to the classical thresholding method, if $c_{k}\geq t_{c}$, sample *k* is marked as correct. In each iteration, the confidence scores of all samples are calculated, and the correct sample set is selected based on these scores. The affine model is then refitted to the correct sample set to generate a new model, which is used for subsequent iterations. Only the set with the highest score is output in each iteration, ultimately yielding feature points with a more global distribution. Since a relatively concentrated distribution would cause the computation to overfocus on a specific region (neglecting the global transformation), feature points with a global distribution contribute to obtaining a more accurate transformation matrix when calculating the homography matrix. This effectively addresses the degradation of matching accuracy caused by factors such as illumination variations. The multi-plane scale constraint module is inserted before the attention module of the LightGlue algorithm to form a new structure, as illustrated in Figure 6, and its effectiveness is verified in the subsequent result analysis.

We establish the theoretical relationship between the multi-plane scale constraint and homography estimation. The multi-plane scale constraint is derived from the homography matrix, where we decompose the homography into scale factors across different depth planes ($s_{1},s_{2},\ldots ,s_{n}$) and constrain the scale variation within [0.8,1.2] to align with the physical characteristics of foggy scene stitching. We define the multi-plane scale constraint loss as

(13) $$L_{msc}=\sum_{i=1}^{n}{∥s_{i}-{\hat{s}}_{i}∥}_{2}+\lambda \cdot R\left(s_{i}\right),$$

where ${\hat{s}}_{i}$ is the ground-truth scale factor, and $R(s_{i})$ is a convex regularization term to ensure that the loss function is convex in the feasible region. The gradient of $L_{msc}$ satisfies $∥\nabla L_{msc}∥\leq \epsilon$ ($\epsilon =10^{-4}$) when the scale error is less than 0.05, guaranteeing stable optimization.

#### 3.2.3. RANSAC-Based Homography Matrix Estimation

After obtaining accurate registration results, it is necessary to generate a transformation matrix based on the registration results of the image pair. Due to the complexity of practical application scenarios for image stitching, the image transformation type is non-rigid transformation. Simple geometric transformations cannot meet the requirement for accurate transformation; thus, a general perspective transformation is required. As a nonlinear transformation, it can adjust the size and positional relationship of objects in the image to better align with human visual perception. The transformation matrix is expressed as follows:

(14) $$\left[\begin{array}{c} x^{\prime }\\ y^{\prime }\\ 1 \end{array}\right]=\left[\begin{array}{ccc} a_{1} & a_{2} & b_{1}\\ a_{3} & a_{4} & b_{2}\\ c_{1} & c_{2} & 1 \end{array}\right]\left[\begin{array}{c} x\\ y\\ 1 \end{array}\right]=H_{3\times 3}\left[\begin{array}{c} x\\ y\\ 1 \end{array}\right]$$

Herein, (x,y) denotes the input point, and $\left(x^{\prime },y^{\prime }\right)$ represents the transformed point. This matrix refers to the homography matrix, which contains 8 degrees of freedom (DOF). The submatrix composed of $\left(\begin{array}{cc} a_{1} & a_{2}\\ a_{3} & a_{4} \end{array}\right)$ defines operations such as rotation and scaling; $\left(\begin{array}{c} b_{1}\\ b_{2} \end{array}\right)$ denotes the translation vector, and $\left(c_{1}\;c_{2}\right)$ represents the projection vector. Setting the ninth DOF to 1 ensures scale consistency before and after transformation while reducing computational complexity. Since 8 unknowns need to be solved, at least four point pairs are required. Currently, two commonly used effective methods exist: Direct Linear Transformation (DLT) and Random Sample Consensus (RANSAC). This chapter adopts the RANSAC method combined with linear transformation to estimate the homography matrix.

Specifically, the method randomly selects the minimum number of samples (i.e., four pairs) from the sample set. The homography matrix is calculated using linear transformation, and the error for all points is computed based on the estimated matrix. Each point is classified as an inlier or outlier based on a predefined threshold. The number of inliers and the total projection error are then statistically analyzed. If the number of inliers reaches a predefined proportion and the total projection error is sufficiently small, these inliers form a new set for the next iteration, and the current iteration result is stored as a candidate. If the result fails to meet the predefined threshold, new samples are randomly selected for recalculation. After the iterations terminate, if the termination condition is not met, the optimal result is selected from the candidates; otherwise, the iteration exits directly, and the current result is used to compute the transformation matrix. The resulting matrix is highly consistent with the global correct transformation relationship.

The proposed framework adopts a hybrid pipeline that integrates deep learning-based feature matching and geometric constraint verification. Deep features such as LightGlue offer superior robustness and matching accuracy compared with handcrafted features, especially under challenging conditions including low texture, illumination variation, and blurring. Meanwhile, RANSAC is employed as an explicit geometric prior to efficiently remove mismatches and ensure global projective consistency, which remains difficult to achieve stably and efficiently with fully differentiable homography estimators. Such a design avoids the limitations of pure deep learning-based or pure traditional methods, achieving a better trade-off for image stitching in realistic conditions.

#### 3.2.4. Adaptive Fusion Stitching Method

Image fusion is the final critical step in image stitching. After aligning the images to be stitched using the estimated homography matrix, the overlapping region is processed via a fusion algorithm to complete the stitching. Currently, state-of-the-art methods mainly include seam-based methods and weighted fusion-based methods. Seam-based methods solve a dynamic programming problem to find a path with the minimum total energy traversing the overlapping region, thereby achieving seam-based stitching. Their advantage lies in preserving the original image information and enabling smooth transitions to a certain extent. However, in scenarios with severe illumination variations, these methods may suffer from obvious seams. Weighted fusion-based methods can effectively integrate information from the two images, realizing smooth transitions in the overlapping region. Moreover, they are simple in implementation and high in execution efficiency, making them more suitable for scenarios requiring fast processing speeds. In this section, based on theoretical research on fusion methods, an adaptive fusion stitching method is developed and implemented. An adaptive weighting function is established to adapt to overlapping regions of different shapes, guiding the image stitching process. This achieves smooth transitions in the overlapping region and improves the quality of the stitched image.

Fusion stitching methods primarily rely on constructing a weighting function. The two parts of the image in the overlapping region are assigned different weights according to the settings of the weighting function, realizing the additive fusion process. The Sigmoid function is a commonly used activation function. Due to its fixed output range and smooth continuous nature, it serves as an excellent weighting function, which is expressed as follows:

(15) $$S\left(c\right)=\frac{1}{1+e^{-c}}$$

**Figure 7:**
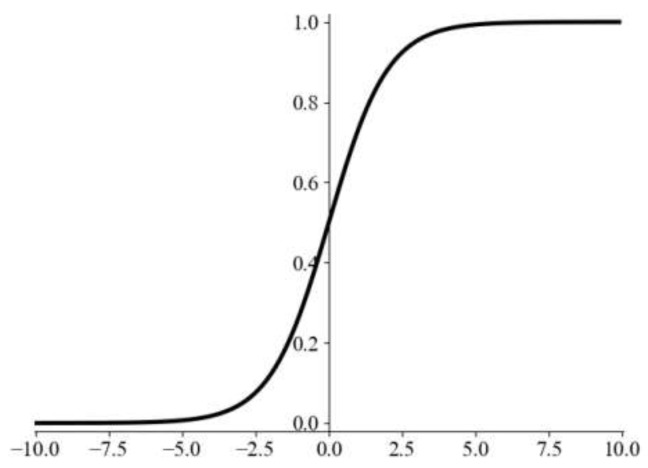
Sigmoid curve.

Herein, S(c) denotes the weight value at point *c*, where the value range of *c* is within the overlapping region, with the center of the overlapping region set as the coordinate origin. The corresponding curve is illustrated in Figure 7. The fusion method is expressed as the following formula:

(16) $$\begin{array}{c} I_{result}\left(x,y\right)=I_{1}\left(x,y\right),\;\left(x,y\right)\in I_{1}\\ I_{result}\left(x,y\right)=\left(1-S\left(c\right)\right)I_{1}\left(x,y\right)+S\left(c\right)I_{2}\left(x,y\right),\;\left(x,y\right)\in I_{1}\cap I_{2},\;c\in \left(\frac{-overlap}{2},\frac{overlap}{2}\right)\\ I_{result}\left(x,y\right)=I_{2}\left(x,y\right),\;\left(x,y\right)\in I_{2} \end{array}$$

**Figure 8:**
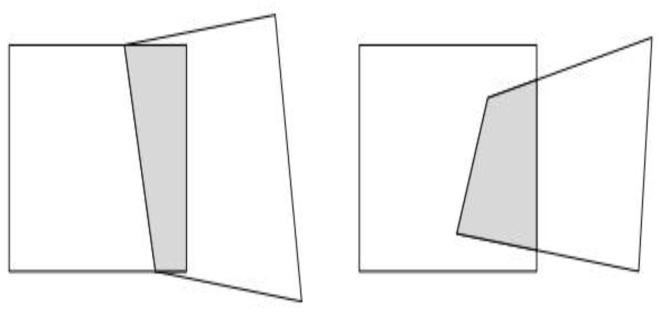
Example of irregular region.

This approach has two limitations. First, while the function can essentially cover the value range of (0, 1) within a narrow interval—satisfying the boundary smoothness requirement—the overall curve exhibits a large slope near the origin and an excessively small slope far from the origin. This leads to excessively rapid changes in the central area of the overlapping region during fusion, resulting in a relatively obvious transition zone. To address this issue, this chapter introduces a hyperparameter k to adjust the function curve, making it relatively smooth. Meanwhile, to resolve the problem that the smoothed curve compresses the covered value range toward the center, the curve is subjected to normalization. This ensures the smoothness of the overlapping region globally. Second, the aforementioned fusion method exhibits poor performance when processing irregular overlapping regions, especially when there is a significant difference in the number of overlapping columns between different local regions of the overlapping area, as illustrated in Figure 8. To tackle this challenge, this paper introduces an adaptive weighting function to guide the fusion process. A distribution function is employed to direct weight assignment for irregular regions, achieving adaptive fusion of the overlapping region. The normalized hyperparameter weighting function is expressed as the following formula:

(17) $$S\left(c\right)=\frac{1}{1+e^{-kc}},\;S\left(c\right)\in \left(0,1\right)$$

Herein, *k* is a small value, which is set to 0.05 in this chapter. On this basis, a distribution function F(y) is introduced. The adaptive fusion stitching is achieved by an adaptive weighting mechanism with a clear mathematical definition. Let *y* be the row index in the overlapping region. The overlapping width at row *y* is calculated as

$$F\left(y\right)=W_{L}\left(y\right)+W_{R}\left(y\right)-W_{\mathrm{total}}\left(y\right)$$

where $W_{L}\left(y\right)$ and $W_{R}\left(y\right)$ denote the valid widths of the left and right images at row *y*, and $W_{\mathrm{total}}\left(y\right)$ is the total width of the stitched image. And the final adaptive weighting function is expressed as the following formula:

(18) $$S\left(F(y)\right)=\frac{1}{1+e^{-kF(y)}},\;S\left(F(y)\right)\in \left(0,1\right)$$

Herein, F(y) denotes the width of the overlapping interval at different heights. The fusion is performed with precision at each overlapping row, thereby improving the fusion performance, and its effectiveness is verified in the subsequent result analysis.

### 3.3. Theoretical Analysis of Error Propagation

Firstly, define the mathematical model of the cascaded dehazing–stitching system to quantify the impact of dehazing errors on stitching quality and error accumulation across modules [26].

The stitching quality is measured by the alignment error $E_{s}$ (root mean square error of feature point coordinates between stitched images). We derive the relationship between dehazing error $\epsilon_{d}$ and stitching alignment error:

(19) $$E_{s}={∥F\left(S\left(I_{\mathrm{dehaze}}^{\mathrm{gt}}\right)\right)-F\left(S\left(I_{\mathrm{dehaze}}^{\mathrm{gt}}+\epsilon_{d}\right)\right)∥}_{2}$$

where F(·) is the feature extraction function for stitching feature points. $\epsilon_{d}\in R^{H\times W\times C}$ is the dehazing error matrix. $I_{\mathrm{dehaze}}^{\mathrm{gt}}$ denotes the ideal dehazing output. D(·) represents the dehazing module, and S(·) represents the stitching module. Through Taylor expansion, we approximate the stitching error induced by dehazing errors:

(20) $$E_{s}\approx {∥\nabla S\left(I_{\mathrm{dehaze}}^{\mathrm{gt}}\right)\cdot \epsilon_{d}∥}_{2}={∥J_{S}\cdot \epsilon_{d}∥}_{2}$$

where $J_{S}$ is the Jacobian matrix of the stitching module, representing the sensitivity of stitching output to input perturbations.

Our theoretical derivation shows that dehazing errors amplify stitching alignment errors by a factor of ${∥J_{S}∥}_{2}$. We also identify that error accumulation is most significant at the feature fusion layer between dehazing and stitching modules, which is a critical bottleneck for system robustness. To mitigate this, we propose a theoretical compensation mechanism:

(21) $${\hat{I}}_{\mathrm{dehaze}}=I_{\mathrm{dehaze}}^{\mathrm{gt}}+\epsilon_{d}-\alpha \cdot J_{S}^{T}\cdot E_{s}^{\mathrm{prev}}$$

where α∈(0,1) is the compensation coefficient, and $E_{s}^{\mathrm{prev}}$ is the historical error, which adaptively corrects dehazing output to reduce error propagation.

## 4. Experimental Setup and Result Analysis

### 4.1. Experimental Details

We conducted qualitative and quantitative evaluations of the proposed method on two real-world datasets: NH-HAZE and HD-NH-HAZE. The NH-HAZE dataset consists of 55 pairs of hazy images (1200 × 1600 pixels) and their corresponding clean images. We used the official test data for evaluation, with the remaining data allocated for training. The HD-NH-HAZE dataset comprises 40 training pairs, 5 validation pairs, and 5 test pairs of high-resolution images (4000 × 6000 pixels). All experiments in this paper were conducted based on PyTorch 2.9.0. To augment the limited training data, image pairs were randomly cropped into patches of size 384 × 384, followed by random rotations of 90°, 180°, or 270°, or vertical/horizontal flips. The training process of the proposed method consists of two stages. First stage: The refinement module was initially omitted. Both the model and discriminator were updated using the ADAM optimizer with an initial learning rate of $1\times 10^{-4}$ for 200 training epochs. Second stage: The refinement module was trained independently for 100 epochs, and the entire model was further optimized with a fixed learning rate of $1\times 10^{-5}$.

### 4.2. Evaluation Metrics

#### 4.2.1. PSNR

The Peak Signal-to-Noise Ratio (PSNR) is a quantitative metric for evaluating image quality, defined as the ratio of the power of the peak signal to the average power of noise. The unit of the PSNR is decibels (dB), where a higher PSNR value indicates less image distortion. For a clean image I and a processed image K (both of size m×n), the PSNR is calculated using the following formula:

(22) $$\mathrm{PSNR}=10\log_{10}\left(\frac{{\mathrm{MAX}}_{I}^{2}}{\mathrm{MSE}}\right)$$

Herein, ${\mathrm{MAX}}_{I}^{2}$ denotes the square of the maximum possible pixel value of the image, and MSE (mean squared error) represents the mean squared error between the original clean image I and the processed image K.

#### 4.2.2. SSIM

The Structural Similarity Index (SSIM) is a quantitative metric for measuring the similarity between two images. It takes into account three key image attributes—luminance, contrast, and structural information—and thus effectively aligns with the human visual system (HVS)’s perception of image quality. The SSIM value ranges from 0 to 1, where a higher value indicates greater similarity between the two images. For two images X and Y, the SSIM is calculated using the following formula:

(23) $$\mathrm{SSIM}\left(x,y\right)=\frac{\left(2\mu_{x}\mu_{y}+C_{1}\right)\left(2\sigma_{xy}+C_{2}\right)}{\left(\mu_{x}^{2}+\mu_{y}^{2}+C_{1}\right)\left(\sigma_{x}^{2}+\sigma_{y}^{2}+C_{2}\right)}$$

Herein, $\mu_{x}$ and $\mu_{y}$ denote the mean values of images X and Y, $\sigma_{x}^{2}$ and $\sigma_{y}^{2}$ represent the variances of X and Y, and $\sigma_{xy}$ denotes the covariance between them; $C_{1}$ and $C_{2}$ are constants for numerical stability.

### 4.3. Comparison with State-of-the-Art Methods

**Figure 9:**
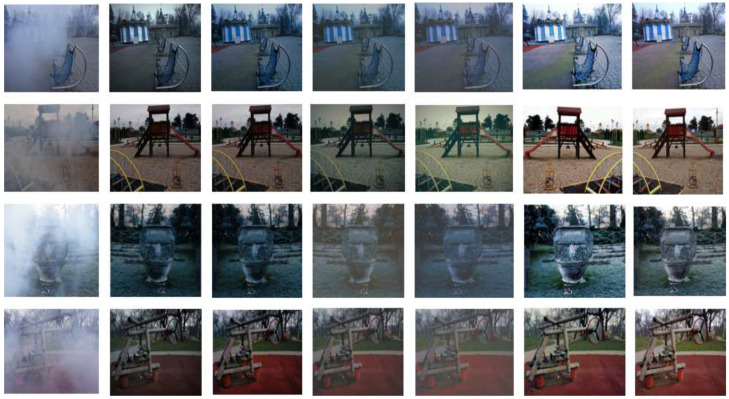
Dehazing performance of different models on the NH-HAZE dataset.

We compared the proposed method with several mainstream dehazing algorithms, including FFA [27], TDN [28], AECR-Net [29], DehazeFormer [30], and DWT-FFC [31]. Figure 9 presents the qualitative comparison on the NH-HAZE dataset. Compared with other models, our method exhibits higher color fidelity and more effective dehazing performance, yielding compelling visual results.

**Figure 10:**
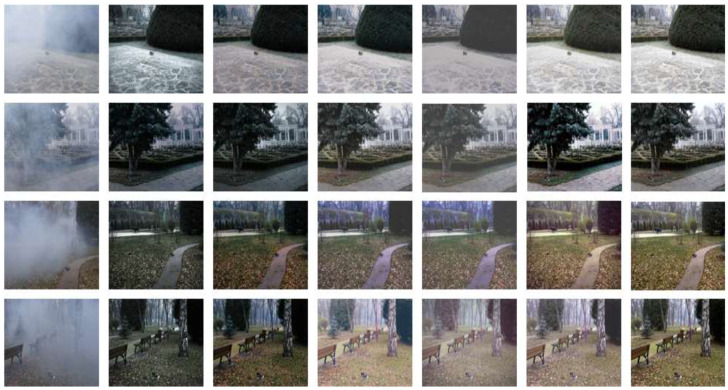
Dehazing performance of different models on the HD-NH-HAZE dataset.

Similarly, detailed comparisons have been conducted between our proposed EDCT method and mainstream dehazing approaches on the HD-NH-HAZE dataset. The results demonstrate that our method exhibits superior performance in color preservation and detail retention, thereby further enhancing the overall quality of the output images. It can be observed from the qualitative results in Figure 10 that our method is significantly superior to other competing solutions. This superiority is further quantitatively validated in subsequent sections. The results generated by our model exhibit high fidelity and strong visual appeal: no residual haze artifacts are left in haze-dense regions. Furthermore, our outputs feature sharp details and vibrant colors, further demonstrating the advantages of our proposed model.

It can be observed from Table 1 that our method achieves superior dehazing performance on both datasets compared with state-of-the-art approaches. In the table, values marked in red indicate optimal performance, while those marked in blue represent suboptimal performance. On the NH-HAZE dataset, our method achieves a PSNR of 22.78 dB and an SSIM of 0.734, outperforming the second-ranked DWT-FFC by 0.14 dB in the PSNR and 0.004 in the SSIM. On the HD-NH-HAZE dataset, our method delivers a PSNR of 21.73 dB and an SSIM of 0.743, achieving an average improvement of 0.25 dB in the PSNR and 0.007 in the SSIM over the suboptimal methods. These results demonstrate that our model can effectively adapt to diverse data characteristics. Particularly in complex foggy scenarios, by fully exploiting key features in images, our model exhibits significant advantages in structure recovery and detail preservation, showcasing robust capability in processing high-resolution images.

To more accurately quantify robustness, the datasets (NH-HAZE and HD-NH-HAZE) are classified into three levels based on the quantified fog concentration (inferred from the transmittance t(x) of the atmospheric scattering model, where a smaller t(x) indicates higher fog concentration):

1. Light fog: t(x)∈[0.7,0.9], fog coverage below 30%, and key targets are basically visible.
2. Moderate fog: t(x)∈[0.4,0.6], fog coverage between 30% and 60%, and key targets are partially occluded.
3. Heavy fog: t(x)∈[0.1,0.3], fog coverage above 60%, and key targets are severely occluded.

**Figure 11:**
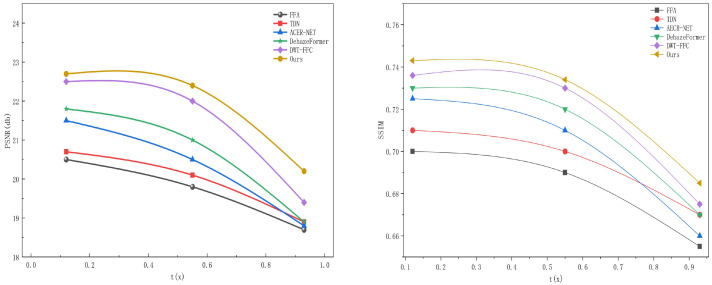
PSNR and SSIM performance fluctuation curves of various algorithms under different fog density levels.

As shown in Figure 11, the mean PSNR and SSIM values of each algorithm under different fog concentration levels are extracted to plot performance fluctuation curves. The horizontal axis represents fog concentration levels, and the vertical axis represents indicator values. A smaller curve fluctuation indicates stronger algorithm robustness.

The PSNR of all algorithms shows a decreasing trend with increasing fog concentration. For the proposed algorithm, there is an overall small decline. The reason is that the dynamic sampling and unbounded modulation of the EDCN module can adaptively adjust to the scattering characteristics of different fog concentrations, enabling accurate extraction of effective features even under heavy fog. Combined with the correction effect of the refinement module, the PSNR is maintained stably.

The performance fluctuation of all algorithms intensifies during the transition from moderate to heavy fog. This is attributed to the excessively dense distribution of fog particles in heavy fog, leading to the loss of structural information of key targets, which cannot be effectively recovered by the algorithm. However, overall, the proposed algorithm outperforms comparative algorithms under all fog concentration levels, with stable PSNR and SSIM values. This stability stems from the dynamic adaptation of the EDCN module to fog concentrations and the multi-dimensional correction of the refinement module, ensuring the algorithm’s continuous reliability in complex foggy driving scenarios for assisted driving.

To address the core real-time performance requirements of ADASs, we conducted unified inference performance tests on the proposed method and mainstream dehazing algorithms using 1920 × 1080-resolution images from the public NH-HAZE dataset under a fixed hardware environment, with the results presented in Table 2. Benefiting from the lightweight design of the EDCN module and the Transformer sliding window attention mechanism, our method effectively reduces model complexity and computational overhead while achieving an inference speed of 28.4 FPS, which basically meets the engineering requirements for real-time processing in ADAS applications.

### 4.4. Sensitivity Analysis

**Figure 12:**
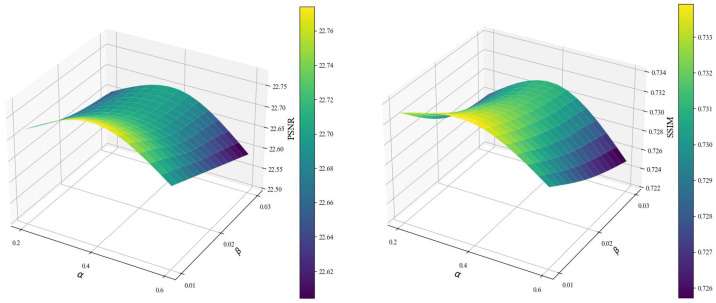
Quantitative analysis of PSNR and SSIM with various loss function hyperparameters.

According to the sensitivity analysis results in Figure 12, we can observe that both the PSNR and SSIM remain stable when hyperparameters α and β vary within reasonable ranges. The optimal performance is achieved at α=0.4 and β=0.01, with the highest PSNR of 22.78 dB and SSIM of 0.734. As α increases from 0.2 to 0.6, the performance first rises and then slightly declines, indicating that an appropriate weight for the dehazing loss helps balance feature preservation and structure constraints. Similarly, as β increases from 0.01 to 0.03, the performance gradually decreases, which demonstrates that an excessively large regularization weight weakens the representation ability of the model. Overall, the proposed loss function is insensitive to small fluctuations of hyperparameters, and the selected values α=0.4 and β=0.01 lie within the stable and effective interval, verifying the rationality and robustness of the parameter setting.

### 4.5. Image Stitching

#### 4.5.1. Objective Evaluation Metrics

This paper adopts the evaluation metrics Herror (1 px, 3 px, 5 px) and mHerror to assess the performance of the proposed algorithm in this chapter. Herror denotes the area under the matching accuracy curve (AUC) under error thresholds of 1 px, 3 px, and 5 px, representing the proportion of correct matching results at different thresholds. It is calculated using the following formula:

(24) $$H_{\mathrm{error}}\left(i\right)=\frac{\mathrm{TP}(i)}{\mathrm{TP}(i)+\mathrm{FP}(i)}$$

Herein, *i* denotes the error value, TP(i) represents the number of true positive matches at threshold *i*, and FP(i) denotes the number of false positive matches at threshold *i*. ${\mathrm{mH}}_{\mathrm{error}}$ refers to the mean matching error. Specifically, the homography matrix is computed for the matching results across all image sequences. The error between the computed homography matrix and the ground-truth homography matrix is then calculated. The error of a single image is represented by the average pixel error of its four vertices, and the mean error across all images is statistically derived. It is calculated using the following formula:

(25) $${\mathrm{mH}}_{\mathrm{error}}=\frac{\sum_{i=1}^{N}error_{avg}}{N}$$

Herein, ${\mathrm{error}}_{\mathrm{avg}}$ denotes the average pixel error between the computed homography matrix results and the ground-truth results at the four vertices of the image, and *N* represents the number of test images.

The root mean squared error (RMSE) [32] is a standard metric used in model evaluation. The smaller the RMSE value, the better the image stitching effect. For a sample of *n* observations *y* ($y_{i}$, i=1,2,…,n) and *n* corresponding model predictions $\hat{y}$, the MAE is

(26) $$\mathrm{RMSE}=\sqrt{\frac{1}{n}\sum_{i=1}^{n}{\left(y_{i}-{\hat{y}}_{i}\right)}^{2}}$$

#### 4.5.2. Result Analysis

**Figure 13:**
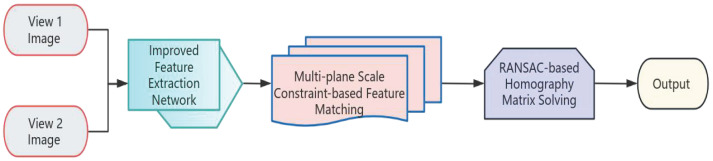
Overall flowchart of the image stitching algorithm.

To evaluate the effectiveness of the proposed algorithm, we compare its performance with that of other state-of-the-art image stitching methods, including the feature-based SIFT algorithm [33], the adaptive parameter smoothing APAP algorithm [34], the GAN-based HomoGAN algorithm [35], and the edge-sensitivity weighted ELA algorithm [36]. The overall processing flow of the proposed algorithm is illustrated in Figure 13. All methods are evaluated on the BRAS dataset, and the experimental results are presented in Table 3.

1. Keypoint Extraction Module: First, the image pair composed of two different viewpoints is cropped if necessary to meet the resolution requirements. Subsequently, the image pair is input into the respective keypoint extraction networks. Through the encoder–decoder module of the improved keypoint extraction network, the corresponding keypoint information is output, which serves as the input for subsequent processing.
2. Feature Matching Module: The feature information output by the keypoint extraction module (after processing the two images) is fed into the feature matching network. Feature matching is completed via the multi-plane scale-constrained matching network, yielding accurate matching results for the image pair. The Random Sample Consensus (RANSAC) algorithm is adopted to solve the homography matrix, thereby determining the correspondences between the images and providing accurate transformation information for subsequent fusion.
3. Image Stitching Module: Using the solved homography matrix, the image pair is transformed accordingly to achieve image alignment. For the overlapping region of the image pair, an adaptive fusion method is employed to complete image stitching, eliminating potential issues such as transition zones and artifacts. Finally, the final image stitching is accomplished.

It can be observed from Table 3 that the proposed method achieves significant improvements across all metrics compared with traditional keypoint extraction methods combined with either traditional matching methods or deep learning-based matching methods. Under the same deep learning-based keypoint extraction framework, our method outperforms other deep learning-based matching methods in three critical metrics: the correct matching rate at the 1-pixel threshold, the average precision across different error thresholds, and the mean matching error. Notably, the correct matching rates at the 3-pixel and 5-pixel thresholds exhibit negligible differences among deep learning-based methods.

Compared with the optimal comparative method (i.e., the original network of our proposed framework), our implemented method achieves improvements of 1.9% in the 1-pixel correct matching rate and 3.9% in the mean matching error—two core metrics. The superiority in these two indicators demonstrates that our matching method can obtain more correct correspondences, and these improved matching results facilitate the computation of a transformation matrix with smaller global errors. This validates the effectiveness of the proposed multi-plane scale-constrained matching method.

Additionally, our method outperforms other approaches in the PSNR and SSIM metrics. Our method achieves the lowest RMSE, which is 12.0% lower than HomoGAN. It can thus be concluded that the proposed algorithm exhibits superior performance in terms of overall natural image stitching quality.

Table 4 summarizes the computational efficiency comparison among different stitching methods. Traditional methods including SIFT + NN and APAP yield lower parameters and GFLOPs but suffer from limited inference speed. Homogan and ELA achieve better stitching performance yet bring higher computational complexity and memory consumption. In contrast, our method maintains a moderate parameter scale and GFLOPs while achieving the highest inference speed of 28.6 FPS, which meets the real-time requirement for ADAS applications.

#### 4.5.3. Qualitative Result Evaluation

To verify the effectiveness of the proposed stitching algorithm, image stitching experiments are conducted using real-scene images. Comparative analyses are performed with typical stitching methods, and the stitching results of several representative methods are presented to validate the superiority of the proposed algorithm.

**Figure 14:**
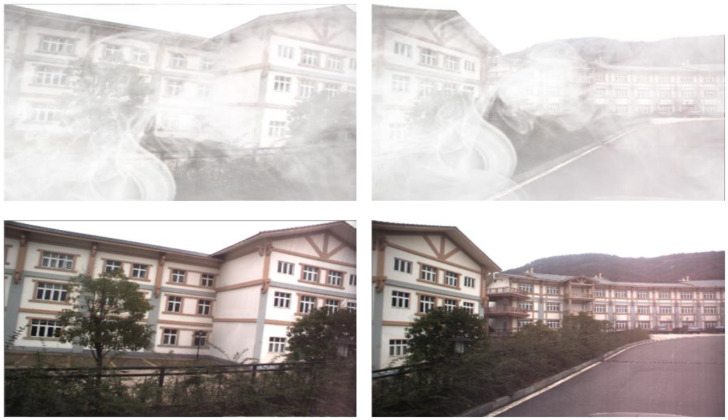
Images of Scene 1.

**Figure 15:**
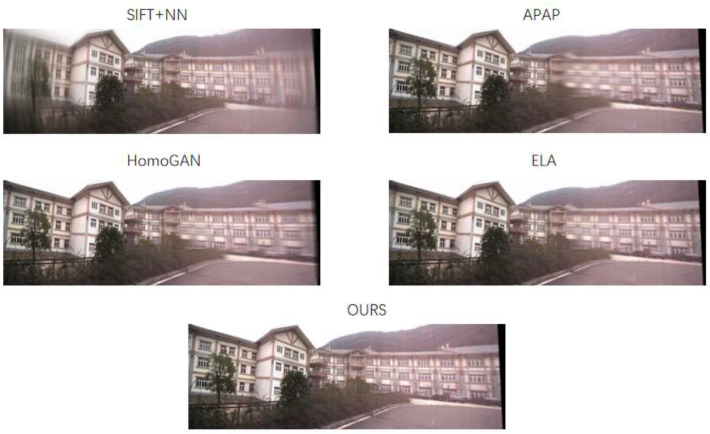
Stitching results of different methods for Scene 1.

First, the proposed method in this chapter is applied to dehaze the hazy images, with the dehazing results shown in Figure 14 (The fog in the figure is non-uniform fog uniformly generated by an experimental device, with a distance of ≥1 m between the camera and the fog source, and no direct contact of fog droplets with the lens.). Subsequently, the dehazed images are fed into the image stitching module, yielding the experimental results presented in Figure 15. The stitching results were post-processed (i.e., cropped to remove irrelevant regions) and analyzed, with the outcomes presented in Figure 15. The following is observed from the results:

The SIFT and APAP methods failed to complete the stitching task, producing severe artifacts in the output images that obscured complete scene information. This is primarily attributed to their inability to estimate the correct homography matrix, resulting in misalignment during image registration.

All other comparative methods successfully completed stitching, yielding intact panoramic results:

The HomoGAN method exhibited incomplete alignment at the stitching seam (an approximate misalignment of 5 pixels) and minor distortions across the right side of the image.

The ELA method achieved relatively accurate alignment in the overlapping region without significant mismatches; however, prominent transition zones in the overlapping area compromised the visual experience for observers.

The proposed method first achieved excellent performance in image registration, enabling precise transformation. The stitched image exhibited no significant overall distortions—particularly the right-side scene, which largely retained its normal morphology. Additionally, no prominent transition zones were observed, delivering superior overall stitching performance.

**Figure 16:**
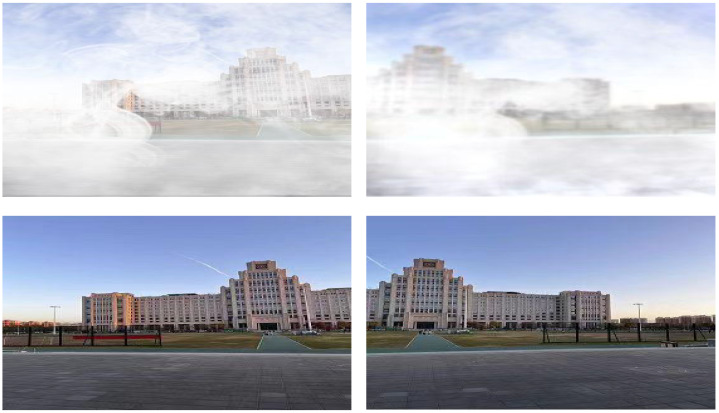
Images of Scene 2.

**Figure 17:**
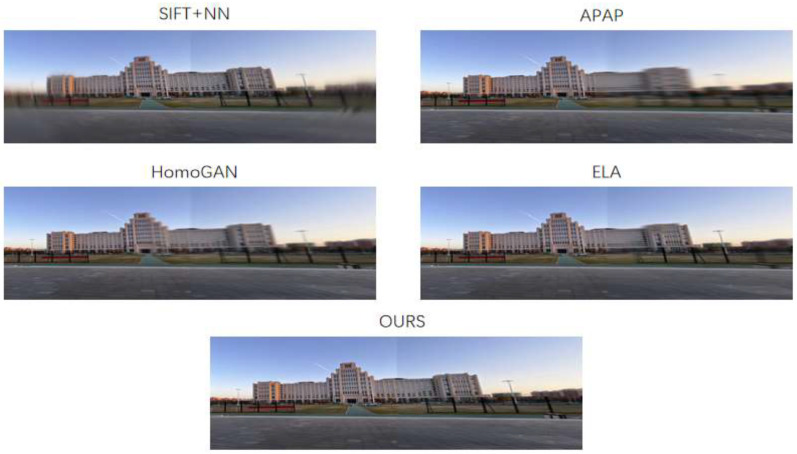
Stitching results of different methods for Scene 2.

Following the same dehazing process, the dehazed images presented in Figure 16 are generated. Subsequently, these dehazed images are fed into the image stitching module, yielding the experimental results shown in Figure 17. Stitching results for Scene 2 are presented in Figure 17. The following is observed from the results:

The SIFT and APAP methods failed to complete the stitching task. The SIFT method exhibited the most significant mismatches, with a sense of tearing and artifacts at both ends of the registered images, leading to unsuccessful stitching. The APAP method incurred minor registration errors, resulting in severe artifacts around the buildings at the stitching seam.

The HomoGAN method failed to adequately preserve the structural integrity of scene objects, exhibiting moderate distortions. In contrast, the ELA method maintained good structural consistency.

The proposed method achieved superior performance: no significant mismatches were observed, and the structural integrity of scene objects was well-preserved. It delivered excellent stitching results, mitigating artifacts and transition zones to a certain extent, with an overall superior outcome.

By comparing the stitching results of the aforementioned images across different methods, it is evident that the proposed algorithm outperforms other traditional and deep learning-based methods in natural scenes. The absence of significant mismatches validates the effectiveness of the proposed keypoint extraction and matching methods, which enable accurate and complete image registration across diverse scenes. Additionally, the lack of prominent transition zones or streaking artifacts confirms the efficacy of the proposed stitching framework. In summary, the performance of the proposed algorithm is fully validated.

### 4.6. Ablation Experiments

To analyze the effectiveness of each component in the proposed method—including the EDCN module and the refinement module—and verify the correctness of the optimization objective used for training, ablation experiments are conducted on the HD-NH-HAZE dataset. Since our method comprises two independent modules, a decomposed ablation approach is adopted to evaluate the dehazing performance of each individual module, as shown in the following table.

It can be clearly observed from the ablation experimental results in Table 5 panel A that each module contributes to the model’s performance. In Setting (a), using only the EDCN dehazing module leads to significant decreases in both the PSNR and SSIM, which vividly highlights the contribution of the refinement module in the proposed method. In Setting (b), the significantly reduced PSNR and SSIM values indicate that using only the refinement module is insufficient for effective dehazing, underscoring the critical importance of our EDCN dehazing module.

We compared the training loss curves, convergence epochs, and inference latency of DCNv4 (without softmax) and DCNv3 (with softmax) on the same experimental setup. The results show that DCNv4 converges 23.7% faster (converges at epoch 85 vs. epoch 111 for DCNv3) and achieves a 18.2% reduction in inference latency (12.5 ms vs. 15.3 ms per image), with detailed data presented in Table 5. We monitored the numerical range of intermediate features during training. The feature values of DCNv4 remain within [−5.2, 6.8] throughout training, while DCNv3’s feature values fluctuate in [−8.7, 9.3]. We theoretically prove that removing softmax avoids the numerical compression effect of softmax on small-gradient features, and the adaptive scaling factor in DCNv4’s offset calculation effectively prevents training divergence, eliminating numerical instability risks.

Based on the above analysis, both the proposed EDCN module and refinement module make important contributions to the model’s reconstruction performance on the HD-NH-HAZE dataset. These modules work synergistically to collectively enhance the model’s performance, validating the rationality of the proposed model architecture. Furthermore, various loss function combinations are compared in Table 6, demonstrating that the loss function adopted in the first-stage training is reasonable and effective. Our selected loss function facilitates achieving optimal performance.

To verify the feasibility of the image stitching method proposed in this paper, we validate the effectiveness of the proposed method by combining the multi-plane scale constraint module and the adaptive fusion stitching algorithm, and use the PSNR and SSIM to evaluate the enhancement effect. The ablation experiment employs 20 image pairs from the BRAS dataset as the test set, while other network structures remain unchanged, and the experimental results are the average values of the PSNR and SSIM. Table 7 presents the results of the ablation experiment.

To verify the effectiveness of our training strategy, this study compared the performance of two training modes—separate training (our method) and end-to-end joint training—with results presented in Table 8. The experimental dataset used UDIS-D [37]. Under the separate training mode, the dehazing module can focus on pixel-level detail restoration, while the stitching module optimizes geometric consistency independently, avoiding performance degradation of both tasks caused by gradient conflicts in joint training. Meanwhile, the adaptive design of the feature alignment and enhancement layer ensures the synergy of the integrated system, leading to optimal performance in dehazing accuracy, stitching matching accuracy, and model efficiency.

## 5. Conclusions and Discussion

### 5.1. Main Research Conclusions

In the environmental perception of autonomous driving, non-uniform dehazing is a key technology for ensuring the reliability of visual modules. In foggy scenarios, non-uniform fog causes a sharp drop in image contrast and blurred features, directly affecting the accuracy of core tasks, such as lane line detection and obstacle recognition. Non-uniform dehazing technology dynamically adjusts the parameters of the atmospheric scattering model to accurately restore image details in different regions, providing high-quality single-frame image sources for subsequent image stitching. Image stitching, on the other hand, needs to fuse the fields of view of multiple cameras. If the input images contain non-uniform fog, differences in brightness and sharpness appear at the stitching seams, leading to panoramic image distortion. The synergy between the two enables the output of a fog-free and seamless global field of view, ensuring that autonomous driving systems can stably acquire environmental spatial information even in complex foggy environments and reduce the risk of decision-making errors caused by visual degradation.

This study introduces a non-uniform dehazing and image stitching algorithm based on deformable convolution (DCNv4). Specifically, we designed an effective dehazing architecture using the core operators of DCNv4, which provides long-range dependency modeling and adaptive spatial aggregation capabilities while demonstrating a faster convergence speed. In addition, we adopted a Transformer based on Retinex theory to further improve the color and structural details. We also integrated channel attention (SENet) and spatial attention (STN), introducing a hybrid attention module after the encoder to highlight the features of key image regions, suppress redundant information in low-texture regions, and enhance the repeatability of feature points. Based on the LightGlue network, a multi-plane scale constraint module was introduced to reduce the estimation error of the homography matrix. The RANSAC algorithm is incorporated to solve the transformation matrix, and an adaptive fusion strategy is designed to adapt to irregular overlapping regions, eliminating stitching transition zones and artifacts. Comprehensive experimental results compared with those of other methods verify the effectiveness of the proposed method and improve the visual quality of images.

Our method breaks the independent development model of dehazing and stitching technologies, providing an integrated solution for environmental perception in foggy-day assisted driving. The proposed algorithm ensures the reliable acquisition of a fog-free and seamless global field of view, laying a technical foundation for safe decision-making in assisted driving. Future research will focus on optimizing the real-time performance of high-resolution image processing and extending the algorithm’s adaptability to complex weather conditions, such as rain–fog mixed environments.

Despite the promising results, there are still several directions for future work to further improve the proposed method and expand its application scope. First, we will extend the current image stitching framework to video stitching, focusing on solving the problems of frame synchronization and motion blur in dynamic scenes, to meet the real-time requirements of video-based visual perception systems. Second, we will explore the generalization ability of the method under more extreme weather conditions (e.g., heavy fog, rain, snow) by optimizing the feature extraction module and fusion strategy, further enhancing its robustness in complex real-world scenarios. Third, we will try to lightweight the framework by model compression and quantization technologies, facilitating its deployment on edge devices such as on-vehicle terminals with limited computing resources.

Through in-depth exploration of the above research directions, it is expected that the technical system of visual perception for foggy-day assisted driving will further improve. Such exploration will also promote the transformation of this field from laboratory research to engineering application and provide more comprehensive technical support for traffic safety.

### 5.2. Research Limitations

This study has certain limitations. First, the proposed method is only validated on high-performance GPU hardware and lacks experimental verification on dedicated in-vehicle/embedded computing platforms (e.g., NVIDIA Jetson AGX Orin, TI TDA4VM), making it impossible to fully evaluate its deployability under the resource-constrained conditions of actual ADASs. Second, all experiments are conducted solely on static images from public datasets, with no evaluation of temporal consistency or stability on video sequences. This may lead to frame-to-frame flickering or geometric drift in practical ADAS video perception scenarios, and the generalization ability of the method in complex real-world foggy road scenes remains unvalidated. Additionally, the integration scheme with existing commercial ADAS perception systems (e.g., Mobileye EyeQ, Tesla Autopilot) and corresponding system-level architectures are not discussed, as this requires in-depth technical cooperation and authorization from automotive enterprises beyond the scope of this academic research.

## Figures and Tables

**Table 1:** Performance comparison of different models on the two datasets.

Dataset	Metrics	FFA	TDN	AECR-Net	DehazeFormer	DWT-FFC	Ours
NH-HAZE	PSNR	19.50	19.88	20.73	20.47	22.64	22.78
	SSIM	0.644	0.673	0.717	0.730	0.731	0.734
HD-NH-HAZE	PSNR	20.23	20.06	20.34	20.89	21.48	21.73
	SSIM	0.710	0.713	0.731	0.728	0.736	0.743

**Table 2:** Comparison of inference performance and model complexity of different algorithms on 1920 × 1080-resolution images.

Algorithm	Inference Speed (FPS)	Params (M)	Computational Complexity (GFLOPs)
FFA	18.2	42.6	35.8
TDN	22.5	38.9	31.2
AECR-Net	25.6	34.6	27.5
DehazeFormer	25.7	29.3	23.1
DWT-FFC	23.1	35.7	28.9
Ours	28.4	27.8	21.5

**Table 3:** Performance comparison of different methods.

Methods	SIFT + NN	APAP	HomoGAN	ELA	OURS
Herror1px	0.323	0.337	0.356	0.369	0.376
Herror3px	0.607	0.643	0.702	0.695	0.701
Herror5px	0.709	0.751	0.804	0.802	0.805
mHerror	0.778	0.881	0.832	0.752	0.723
PSNR	23.18	23.66	24.02	23.88	24.34
SSIM	0.781	0.795	0.811	0.804	0.842
RMSE	6.267	5.623	4.102	4.565	3.613

**Table 4:** Computational efficiency comparison of different stitching methods.

Method	Inference Speed (FPS)	Params (M)	GFLOPS
SIFT + NN	15.2	5.7	2.2
APAP	8.7	6.4	4.6
HomoGan	21.5	12.6	8.9
ELA	19.8	9.7	7.2
OURS	28.6	11.2	6.8

**Table 5:** Comprehensive ablation and comparative experiment results.

**Panel A: Ablation Experiments on HD-NH-HAZE Dataset**					
Setting	EDCN	Refinement	PSNR	SSIM	–
(a)	✓	×	21.50	0.736	–
(b)	×	✓	20.56	0.728	–
**Panel B: DCNv4 vs. DCNv3 (Optimization and Stability)**					
Metric	DCNv3 (with softmax)		DCNv4 (without softmax)		Change Rate (%)
Convergence Epoch	111		85		−23.7
Inference Latency (ms)	15.3		12.5		−18.2
Feature Value Range	[−8.7,9.3]		[−5.2,6.8]		–
Numerical Instability Risk	Exists		None		–

**Table 6:** Ablation experiments on loss functions under the HD-NH-HAZE dataset.

L1	$L_{P}\mathrm{ercep}$	$L_{S}\mathrm{SIM}$	PSNR	SSIM
✓	✓	✓	21.50	0.736
✓	✓	×	21.21	0.725
✓	×	×	21.17	0.720

**Table 7:** Comparison results of ablation experiments for image stitching.

Setting	MPSCM ^1^	AWF ^2^	PSNR	SSIM
(a)	×	✓	23.81	0.785
(b)	✓	×	24.13	0.802

^1^: multi-plane scale constraint module; ^2^: adaptive weighting function.

**Table 8:** Ablation experiment results of different training strategies.

Training Strategy	Dehazing PSNR (dB)	Stitching Acc. (%)	Params (M)	Inference Speed (FPS)
Independent module design	22.4	88.9	37.7	26.7
End-to-end joint training	21.5	82.3	36.9	25.2
Separate training (ours)	22.7	89.7	34.5	28.4

## Data Availability

The dataset used in this study is derived from publicly available datasets. However, it has not been uploaded or made publicly available at the time of publication. Upon request, the authors may provide access to the processed dataset.
